# Editorial: Updates on the management of glioblastoma

**DOI:** 10.3389/fonc.2025.1526669

**Published:** 2025-02-05

**Authors:** Dorota Lubanska, Sameh Elmorsy Hassan, Lisa A. Porter, Mohamed A.R. Soliman

**Affiliations:** ^1^ Department of Biomedical Sciences, University of Windsor, Windsor, ON, Canada; ^2^ WE-SPARK Health Institutel, Windsor, ON, Canada; ^3^ Neurosurgery Department, Sheikh Zayed Specialized Hospital, Cairo, Egypt; ^4^ St. Joseph’s Health Care London, London, ON, Canada; ^5^ Lawson Research Institute, London, ON, Canada; ^6^ Neurosurgery Department, Cairo University, Cairo, Egypt; ^7^ Neurosurgery Department, University at Buffalo, Buffalo, NY, United States

**Keywords:** glioblastoma, prognostic markers, immunotherapy, imaging, diagnostic technology, therapeutic efficacy assessment

Glioblastoma (GBM) is the most common and malignant primary brain tumor with an extremely poor median survival time of only 15 months and a dismal 5-year survival of just 7.2% (1). The significant therapeutic challenge, posed by glioblastoma, stems from its genetic and phenotypic heterogeneity fueled by multiple components of the dynamic system of the tumor including treatment-resistant glioma cells and tumor microenvironment (TME). The current standard of care therapy for glioblastoma consists of surgical resection of the tumor followed by chemoradiation and adjuvant chemotherapy using an alkylating agent, temozolomide (2). Studies published to date, demonstrate the complexity of mechanisms by which glioblastoma tumors overcome the pressure of currently established treatments allowing for post-therapy disease progression, tumor recurrence and patient relapse (3). There is a desperate need for novel strategies in detection, diagnostics and targeted therapeutic approaches to attain successful containment of this deadly disease.

The goal of this Research Topic was to consolidate and showcase studies that encompass new approaches to the management of glioblastoma (**Figure 1**). In this editorial, we highlight novel and trending directions in research based on 16 studies of the Research Topic including seven original articles, two minireviews, six reviews, and one case report. The diversity of the content reflects the necessary innovation to seek, analyze and discuss breakthrough findings to advance diagnostic tools, therapy, and specifically, immunotherapy which is the focal point of several articles in the Research Topic and will be the context of this editorial.

## Evaluating emerging prognostic markers and features toward improved prediction of immunotherapy responses

In recent years the importance of the TME in influencing the biology of glioblastoma has been gaining interest. To date, this has been heavily focused on the analysis of infiltrating cells of the immune system, aiding not only in the classification of the tumors but also in assessment for potential immunotherapies (4, 5). In the study by Guo et al., researchers investigated the expression profile of G protein-coupled receptors (GPCRs) in glioblastoma using both publicly available tumor bulk RNA sequencing and in-house single-cell RNAseq to evaluate RNA abundance and association with the TME. The study constructed a risk model associated with GPCR expression and created a TME score, based on infiltrated immune cells. This work uncovered new survival and immune phenotypes which are marked by differential expression of GPCRs and show that patient outcomes correlate positively with GPCR/TME score. Although these results demonstrate a potential benefit in predicting disease progression, recent clinical data shows that the efficacy of immunotherapies is determined not only by the immunogenicity of the tumor but also by the baseline systemic immunological competence of a patient, which remains to be considered in this model.

Interpatient heterogeneity in immunosuppressive activity is reflected in variable dysregulation of diverse components of the immune system including a decrease in T lymphocytes (lymphopenia) or changes in the neutrophil-to-lymphocyte ratio. Assessment of the absolute counts, ratios and activation states of both circulating as well as tumor-infiltrating immune cells result in differences in the responses to therapy, tumor progression, recurrence and survival (6). Tumor-related immunosuppression is further exacerbated by the standard of care, including radio- and chemo-therapy which leads to immunodeficiency in patients with glioblastoma resulting in therapy-induced (iatrogenic) systemic immunosuppression. In the face of failed attempts to identify a reliable prognostic immune marker over the years, two reviews in our Research Topic discuss the prognostic value and critical role of assessing diverse immune system components both at baseline and post-therapy in patients with glioblastoma. Stepanenko et al. calls for incorporation of dynamic assessment of blood inflammatory markers and parameters, including neutrophil-to-lymphocyte ratio (NLR), and post-treatment total lymphocyte count (TLC) as well as steroid use, in neuro-oncology studies and careful evaluation regarding their prognostic significance. Analysis of existing results on standard therapy-promoted immunotoxicity, in another review- “ *Systemic and local immunosuppression in glioblastoma and its prognostic significance* ”, by Stepanenko et al., shows that post-therapy lymphopenia is a prognostic marker for shorter survival in patients with glioblastoma. In addition, collected data demonstrates that radiation-induced decrease in lymphocyte count is exacerbated by treatment using corticosteroids, which causes immunosuppression, and serves as a poor prognostic marker. The authors point to crucial clinical data to collect when designing clinical trials for immunotherapies and conclude that the improving success of immunotherapies depends on changes to the standard-of-care paradigm.

Another critical component to consider is the potential role of sex-dependent biology on glioblastoma tumors. In a minireview by Jovanovich et al., researchers compiled data regarding sex-related differences in glioblastoma biology, reported over the past ten years, and studied potential correlation with pathogenesis as well as treatment response. This review highlights critical differences in mechanisms that may predict overall survival and treatment responses between males and females. This includes imaging radiomics-based signatures, DNA methylation patterns, timing of glioma-driver mutations and differences in metabolic and immune profiles. Females are characterized by a more active adaptive immune system, higher levels of CD4^+^ T cells, and significantly improved overall survival in comparison to males in immunotherapy clinical trials. The studies collected and reviewed by Jovanovich et al. validate the critical importance to personalize approaches and to consider male and female samples separately when profiling glioblastoma tumors.

## Novel tools in the assessment of therapy responses

Change in contrast enhancement which is present in approximately 98% of gliomas and is related to biological processes of the tumor like hypoxia and inflammation, has aided in measuring therapeutic efficacy in patients with glioblastoma (7). Directly assessing responses to immunotherapies in glioblastoma is complicated by a transient inflammatory-based increase in enhancing volume determined as pseudo-progression (PsP) which must be distinguished from true tumor progression (TTP) to avoid the continuation of non-effective therapies (8). This editorial highlights a study by Cuccarini et al. in which treatment response assessment maps (TRAMs) were employed to test application in immunotherapy. The study used dendritic cells to assess the diagnostic value of TRAMs in the distinction of PsP and TTP in glioblastoma. TRAMs exploit the principle of delayed contrast imaging which allowed the researchers to identify areas of early contrast-medium clearance, which reflected tissues of the tumor, with high sensitivity and specificity. They also distinguished these areas from areas of contrast-medium accumulation which were caused by treatment. This is the first pilot study of the application of TRAMs for immunotherapy which demonstrates that TRAMs could constitute an alternative or supplemental tool to differentiate between pseudo- and true tumor progression providing early markers of therapy response.

## Innovative therapeutic combinations

The current standard of care for glioblastoma results in poor clinical outcomes with a significant majority of patients experiencing recurrence/progression of an incurable tumor. There is an obvious need for testing therapeutic strategies which combine the most promising approaches. There is substantial effort occurring to optimize immunotherapies for glioblastoma; these works are summarized in detail along with other current treatment approaches in the general review on treatment advances in high-grade glioma by (Chen X. et al.). In their review, Bartusik-Aebisher et al. discuss the immunomodulatory functions of photodynamic therapy (PDT). PDT is a treatment based on light energy combined with a compatible drug molecule which, when photosensitized, destroys cells which absorbed it. PDT contributes to the activation and influx of immune cells, leukocytes, lymphocytes and macrophages into the tumor tissue, triggering an inflammatory response in addition to the release of antigens from damaged tumor cells. The application of PDT helps with directing the immune response towards the tumor and can be a beneficial strategy combined with standard of care and immunotherapy.

A retrospective study by Wang et al. shows that delivery of low-intensity, low-frequency alternating electrical tumor-treating fields (TTFields) at the tumor site significantly improved progression-free and overall survival in this small cohort of patients. They demonstrated increased effectiveness in patients at post-tumor resection and with methylated MGMT, suggesting that addition of TTFields to the current standard of care may improve clinical outcomes of glioblastoma treatment. In addition, the evidence discussed by Chen X. et al. shows that the application of TTFields maintains the viability of T-cells, promotes phagocytosis of dendritic cells and synergizes with anti-PD-1 therapy in preclinical models, suggesting that the combination of TTFields and immunotherapy can potentially improve clinical outcomes in glioblastoma.

In the mini review by Liu et al. protein lactylation is explored as a potential therapeutic target in the treatment of glioblastoma. Lactate metabolism and levels of lactylation in the tumor microenvironment play a role in recruitment, activation and maintenance of the components of the immune system (9, 10). As the authors discuss, changes to the lactylation levels of specific proteins predict response to immune checkpoint inhibitors, hence combining of the modulation of the lactylation levels of certain proteins with immunotherapy could potentially benefit clinical outcomes of patients with glioblastoma.

## Conclusion

**Figure 1:**
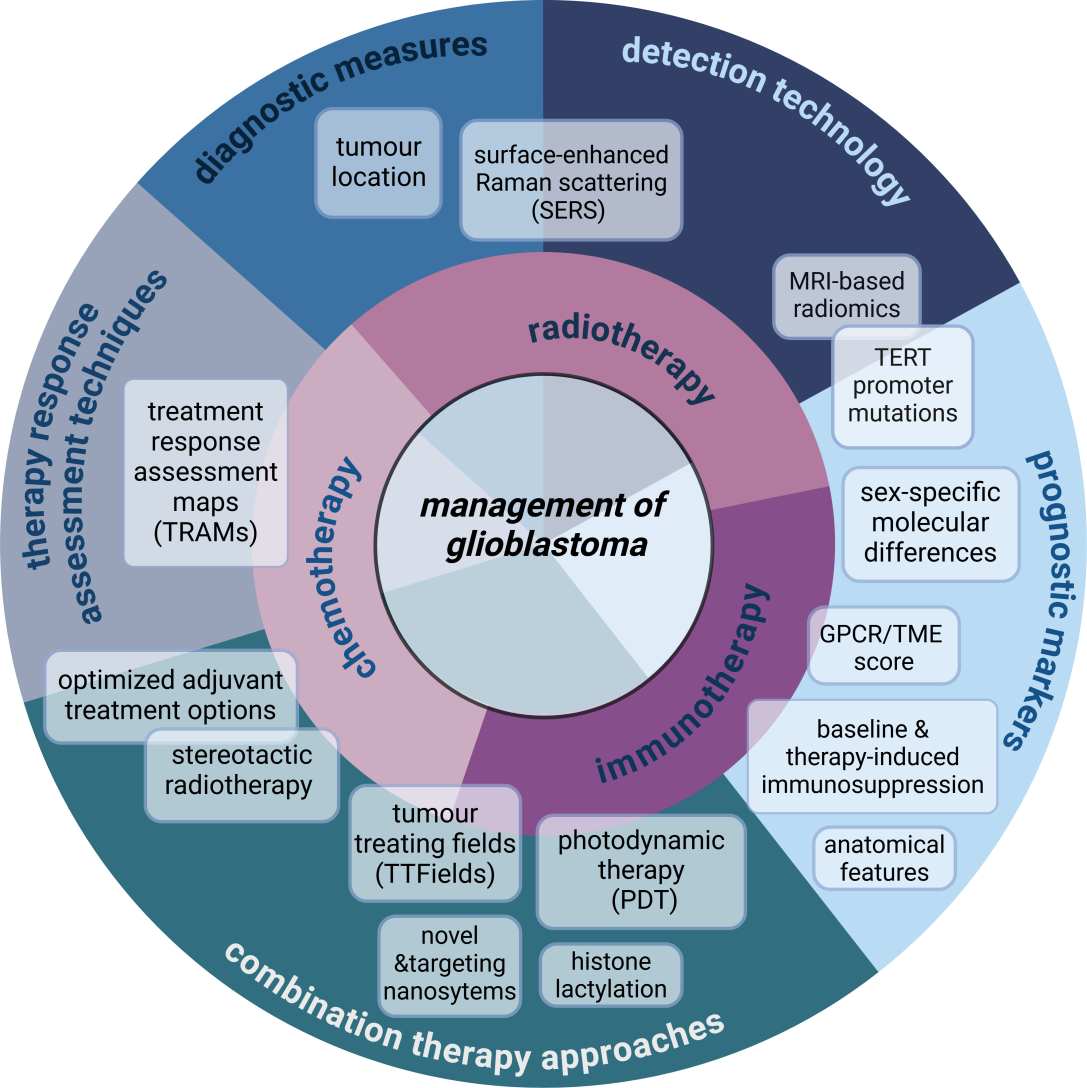
Overview of the specific content and subject areas of the management of glioblastoma included in the Research Topic. This Research Topic showcases a wide variety of studies which discuss novel findings and diagnostic tools related to immunotherapy, but also consolidates new additional data of high importance to the management of glioblastoma. Combination therapy approaches including those utilizing nanosystems are discussed for targeting of glioblastoma recurrence in the case study by Zhong et al., and in the original article by Abu-Serie et al. Zhu et al. employ machine learning models to optimize adjuvant treatment options. Novel diagnostic measures are explored with the use of SERS (Yang et al.) and the analysis of specific anatomical tumor location (Meyer et al.). Chen L. et al. and Tang et al. demonstrate the potential of MRI-based radiomics-mediated detection of TERT mutations and temporal muscle thickness, respectively, as novel prognostic markers, further exemplifying the diversity of the research conducted in the Research Topic. Created in https://BioRender.com.

This Research Topic provides a snapshot of current and emerging strategies for predicting therapy response and enhancing the effectiveness of therapies for glioblastoma (**Figure 1**). While patient outcomes remain dire, research continues to evolve, teaching us more about the complex biology and clinical characteristics of this aggressive disease. Several of the studies in this Research Topic demonstrate the ability to improve effectiveness of immunotherapy, which is currently the fastest growing field with great clinical potential for glioblastoma. This Research Topic highlights the progress happening in the glioblastoma field. Researchers continue to pioneer ways to enhance survival rates and improve patient quality of life. The complexity of glioblastoma necessitates continued efforts to collaborate, to advocate for dedicated funding and patient-centric research to sustain and accelerate progress in this field. Let us unite in focus and determination to deepen our understanding and develop solutions that will have a lasting impact on the lives of glioblastoma patients. Every step forward is a beacon of hope, and together, we can transform the glioblastoma landscape.
